# Notes on Preparations for the Sick

**Published:** 1903-06

**Authors:** 


### Notes on Preparations for the Sick.

**Lofotol**: the new Sparkling Cod Liver Oil.—SOUTHALL BROS. & BARCLAY, Birmingham.—Cod liver oil has been decidedly unpopular of late, and we think it must be very much less used than formerly.

Possibly the introduction of a new and improved preparation may reinstate the drug in its former position as the one thing needful in all tubercular diseases, more especially as it has always been looked upon as a food; and now that feeding is everything, cod liver oil should be a most useful adjunct to the fatty foods taken at meals. The various emulsions and malt combinations have to a very large extent taken the place of the crude oil, but it must be remembered that the dose of the oil when given in these combinations must be very much less than was commonly the rule some ten or fifteen years ago.

This new combination of cod liver oil with carbonic acid gas seems likely to mark a new era in the therapeutics of oleum morrhuæ. The peculiar advantages of Lofotol are: (1) Rapidity of digestion, owing to the stimulating effect of carbonic acid; (2) Ease of digestion, owing to the disintegration of the oil in the stomach caused by the entangled molecules of gas; (3) Freedom from tendency to oxidization and rancidity. The oil is always sweet, and contains little or no free fat acid; (4) Great palatability, owing to the pleasantly sharp and sparkling taste given to the oil by the gas.

From a somewhat limited experience of the combination, we are inclined to think that the proprietors have not claimed too much for it, and that if cod liver oil be needed, this is the variety which is likely to be most acceptable and most useful to the patient.

**Plasmon; Beef Plasmon; Plasmon Diabetic Biscuits.**—INTERNATIONAL PLASMON LTD., London.—The plasmon milk product was mentioned in our *Journal* for June, 1900 (vol. xviii., p. 173). Since that time many combinations have been introduced, and of these the Beef Plasmon, the Plasmon Arrowroot, and the Plasmon Diabetic Biscuits have recently been forwarded. Of the plasmon itself it is needless to say that it is of the highest nutritive value, tasteless, odourless, and easily digested.

Examination of the Beef Plasmon shows the presence of a large amount of proteid, including albumins and globulins, with only a mere trace of salt.

The Diabetic Biscuits are made both plain and with saccharine. They contain no starch and no sugar, but give well-marked proteid reactions. These biscuits are made up of plasmon, pure almond flour, and pure gluten flour. They contain 43 per cent. of assimilable proteid and 5 per cent. of fat. They are useful, not only in cases of diabetes, but in the treatment of obesity.

---

**Casumen**: Prideaux's pure Milk Proteid.—PURE CASEIN AND LIFE FOOD CO. LIMITED, Motcombe, Dorsetshire.

This powder, soluble in water, is separated from milk by a special process, which gives the milk proteid in a pure and soluble state. Mr. Oliver Davis's analysis (University College, Bristol, Laboratory) shows it to be an almost completely soluble proteid: it contains no starch and no sugar. The *Lancet* analysis shows it to contain (when dry) the very large proportion of 93 per cent. of pure proteid. Its food value is therefore very high. It may be added to almost every variety of liquid food, thereby increasing immensely the nutritive value of the fluid; and it is sold in a variety of combinations with arrowroot, macaroni, cocoa, chocolate, and baking powder.

Casumen bread may easily be obtained, also casumen biscuits, and all kinds of pastry, by mixing one or two ounces of dry casumen with a pound of flour.

---

**Hovis Food.**—Hovis Flour Mills, Macclesfield.—Samples of two foods have been forwarded by the Hovis Bread Flour Company:—

No. 1. Infant's food; No. 2. For infants and invalids.

An analysis, made in the University College, Bristol, Laboratory by Mr. Oliver Davis, B.Sc., informs us that No. 1 food consists of proteid, maltose, and dextrine, very rich in phosphates, but with no starch; No. 2 agrees with No. 1 in qualitative composition, but it contains a certain amount of starch.

The detailed analysis issued by the Company is as follows:—

| | NO. 1. | NO. 2. |
|---|---|---|
| Maltose ... ... ... ... ... | 62.41 | 67.49 |
| Dextrine, etc. ... ... ... ... ... | 23.98 | 14.94 |
| Cane sugar ... ... ... ... ... | none | none |
| Nitrogenous compounds ... ... ... | 7.74 | 5.78 |
| Farinaceous matter ... ... ... ... | none | 7.50 |
| Starch cellulose ... ... ... ... | 0.10 | 0.04 |
| Fat ... ... ... ... ... ... | 0.20 | 0.10 |
| Mineral matter (including phosphates) ... | 1.82 | 1.70 |
| Moisture ... ... ... ... ... | 3.75 | 2.45 |
| | 100.00 | 100.00 |
| Soluble in *cold* water ... ... ... | 95.30 | 90.80 |

The small amount of matter insoluble in cold water is freely digestible, leaving the minutest trace (as shown in the above analysis) of cellulose in a soft, light condition.

These foods are well devised, and will doubtless often facilitate the solution of the difficult problems associated with the feeding of infants and invalids.

---

**Vitalia Meat Juice** (Liq. Hæmoglobin Co., c ext. Carnis).—THE VITALIA COMPANY, Blackfriars, London, S.E.—We made some comments on this meat juice in our December number of 1897 (vol. xv., p. 361). Mr. Oliver Davis (University College Laboratory) has recently examined a sample, and he reports that it contains a large amount of proteids, including albumins, globulins, and hæmoglobin; it contains also various extractives, and sodium chloride. It is a palatable preparation of hæmoglobin in combination with Australian extract of meat: its full designation indicates its composition and its nutritive value. It rarely fails to give satisfactory results clinically, and if the colour be an objection to its use, this difficulty may be surmounted by presenting it to the patient in a red-coloured glass.

---

**Vibrona.** — FLETCHER, FLETCHER & Co., London. — This preparation of cinchona was just mentioned in our *Journal* of December, 1895 (vol. xiii., p. 310), and it fully deserves all the reputation it has since received. It is described by the proprietors as a combination of hydrobromic extract of cinchona bark, manufactured under Fletcher's patent of 1894, with a red wine, each wineglassful being equivalent to five grains of cinchona bark. It is an elegant preparation of the bark, which has been found to be of much service as a general tonic, and, in ordinary doses, is not likely to give rise to the symptoms of cinchonism, or to leave any evil after-effects. Dr. B. H. Paul certifies that the wine is what it professes to be, and that it is carefully standardised under his supervision. It claims to be an ideal tonic wine, and experience fully justifies this claim.

---

**Byno-Cascada.** — ALLEN & HANBURYS, London. — Of the various preparations of bynin, this is perhaps likely to be one of the most useful. Being a combination of malt-extract with cascara sagrada and rhamnus frangula, it is something more than a stimulant to peristaltic action. It is called "an ideal tonic laxative," and we think that the results quite justify this appellation. The bynin is an excellent vehicle for the aperient bitter extracts, and the combination must have a good effect on the patient in all cases of portal congestion and atony of the alimentary canal. The treatment of habitual constipation has been much facilitated by the advent of the now familiar cascara, and we have found this preparation to be one of the most efficient of them all.

